# Citizen science in eDNA monitoring for Mediterranean monk seal conservation

**DOI:** 10.1186/s12862-024-02338-8

**Published:** 2024-12-24

**Authors:** Sofia Bonicalza, Elena Valsecchi, Emanuele Coppola, Valeria Catapano, Harriet Thatcher

**Affiliations:** 1 Department of Biomedical Sciences, University of Edinburgh, Edinburgh, UK; 2 Gruppo Foca Monaca APS, Via Carlo Emery 47, 00188 Rome, Italy; 3 Department of Environmental and Earth Sciences, University of Milano-Bicocca, Piazza della Scienza 1, 20126 Milan, Italy

**Keywords:** Conservation, Citizen science, eDNA, Mediterranean monk seal, Social impact

## Abstract

**Background:**

Citizen Science (CS) offers a promising approach to enhance data collection and engage communities in conservation efforts. This study evaluates the use of CS in environmental DNA (eDNA) monitoring for Mediterranean monk seal conservation. We validated CS by assessing the effectiveness of a newly developed CS-friendly filtration system called “WET” (Water eDNA Trap) in eDNA detection, addressing technical challenges, and analysing volunteer faults. The WET is a 4-litre, manual pump-based filtering system using positive pressure to force water through the filter. We also assessed the use of a retrospective questionnaire as a tool to measure CS’s social impact on participants’ perceived knowledge, attitudes, and conservation behaviours.

**Results:**

Results suggest the WET performs comparably to traditional methods, with minor technical issues. Despite some faults such as not folding or forgetting to change the filter, volunteers were generally reliable in sample processing. Moreover, CS involvement increased participants’ perceived knowledge, affective attitudes, and conservation behaviours towards seal conservation. Volunteers reported a greater understanding of eDNA monitoring, increased interest in monk seal conservation, and more frequent conservation behaviours, including spreading awareness within their community. While these findings are exploratory due to the small sample size (19 participants) and potential ceiling effects in attitude assessment, they provide an initial validation of the questionnaire as a tool for measuring CS’s social outcomes. Future studies with larger sample sizes are needed to confirm these results and investigate their applicability across broader stakeholder groups. Continuous improvement in volunteer training and equipment design is also recommended.

**Conclusions:**

This study highlights CS’s potential to improve public engagement and knowledge in conservation. By involving diverse participants, CS can play a critical role in long-term conservation efforts and promote sustainable coexistence between humans and monk seals. Furthermore, the validation of the questionnaire offers a valuable framework for evaluating the social impact of CS initiatives in conservation contexts.

**Supplementary Information:**

The online version contains supplementary material available at 10.1186/s12862-024-02338-8.

## Background

Conservation biology is an interdisciplinary field aiming at protecting biodiversity, rooted in biological sciences and integrated with social sciences [1]. To achieve the conservation of species and ecosystems, ecological monitoring is crucial but not sufficient alone [2], the social aspect must also be considered since it will be human behaviour to eventually determine the success of conservation [3, 4]. Nevertheless, despite the importance of social aspects in conservation being widely recognized, their incorporation is still limited [3].

The Mediterranean monk seal (*Monachus monachus*), hereafter seal, is a priority species for conservation, as despite its recovery it remains one of the world’s most endangered marine mammals, with an estimated global population of 815–997 individuals [5]. It has been exploited by humans since prehistorical times [5, 6]. Currently, the major threats to seal conservation are considered to be marine and terrestrial habitat loss and degradation due to increasing human activity, negative interactions with fishermen such as by-catch and deliberate illegal killings, pollution and unpredictable environmental threats [5]. As threats are human-derived, conservation actions should focus on human interactions, hence the importance of raising awareness and engagement among local stakeholders [7].

Another challenge for seal conservation in the central and western Mediterranean Sea, including Italy, is the significant data deficiency in these areas [8]. Data deficiency poses a critical conservation issue, as it limits our understanding of the population status which is essential for effective conservation planning [9]. To cope with such a problem, environmental DNA (eDNA) monitoring - i.e. collection and analysis of environmental samples such as marine water to detect DNA traces of a target species or taxonomic group [10, 11] - has been identified as a valuable seal monitoring method in low-density and data deficient areas [12, 13]. While eDNA cannot yet provide qualitative or quantitative data on population size or precise behaviors, its ability to detect presence through consistent monitoring can indicate whether an area is frequented regularly or occasionally, making it particularly useful in such contexts.

The eDNA monitoring can be coupled with Citizen Science (CS), which is the involvement of citizens in data collection and other research activities [14], and it has mostly been used for ecological monitoring and to address conservation issues [15]. CS has both scientific and social impacts [16], thus being a powerful tool in biodiversity conservation [17]. The main scientific advantage is to acquire or manage cost-effective data at spatiotemporal scales and resolutions unreachable by researchers alone [18]. Despite the use of CS increasing exponentially, some authors still question CS data reliability and data quality [19, 20]. Nevertheless, it is acknowledged that by following protocols and with appropriate training, volunteers can gather data of quality comparable to that of researchers [19, 21].

The social advantages of CS are multiple and include raising awareness, learning, civic participation, and engagement in conservation [15, 22]. The collaboration between researchers and communities can also help in establishing protected areas and sustainable practices attentive to the well-being of both wildlife and people [16]. Even if each CS project is different and, consequently, will have an evaluation unique to that project, most of them share common goals, including learning outcomes, which are measurable cognitive, affective, and behavioural changes perceptible among the participants [23]. Whilst studies assessing learning outcomes of CS initiatives are still rare [15, 23], results are encouraging and show that CS can be an effective tool in improving people’s knowledge, attitudes or behaviours towards a conservation objective [24, 25]. For a species threatened by human behaviour, such as the seal, CS could become a valuable ally for its conservation, especially by improving the human-seal relationship.

This study uses data collected within Care4Seals, a project by Gruppo Foca Monaca APS (GFM), aiming at combining scientific research with stakeholder’ engagement in seal conservation in the central Mediterranean Sea through CS, public initiatives, training for students, and educational events. The scientific research is integrated with Spot the Monk, an ongoing seal eDNA monitoring campaign launched by the University of Milan Bicocca (www.spot-the-monk-observatory.com). The present monitoring was the continuation of this program, which already involved volunteers in the water sample collection (but not filtration) for eDNA monitoring of the seal. Involving volunteers allows for broader data collection, enhancing spatiotemporal coverage [26, 27]. While previous eDNA studies enlisted volunteers in filtration, they typically handled small volumes (90 ml to 1500 ml) [22, 28–32]. This study, however, required filtering larger water volumes (12 L) to align with the seal eDNA monitoring protocol [13]. As protocols requiring a large volume of water to be transported to a central filtration station or relying on costly electric pumping tools are unsuitable for CS initiatives [30], in this study volunteers used a newly developed CS-friendly device, the WET (Water eDNA Trap), as an alternative to the traditional vacuum pump system.

The overarching aim of this study is to assess the validity of CS associated with eDNA analysis for seal conservation, focusing specifically on the following objectives:

1. Scientific validity: Evaluate the accuracy and reliability of CS in collecting eDNA data, including the performance of the newly developed CS-friendly filtration system (WET), its limitations (e.g., contamination risks), and the faults made by volunteers during the eDNA collection and filtration processes; and
2. Social impact: assess the effectiveness of a retrospective questionnaire in measuring CS’s social impact on participants’ perceived knowledge, attitudes, and conservation behaviors regarding monk seals, and test whether significant changes occur before and after their involvement.

## Methods

### Study area and volunteers

**Fig. 1:**
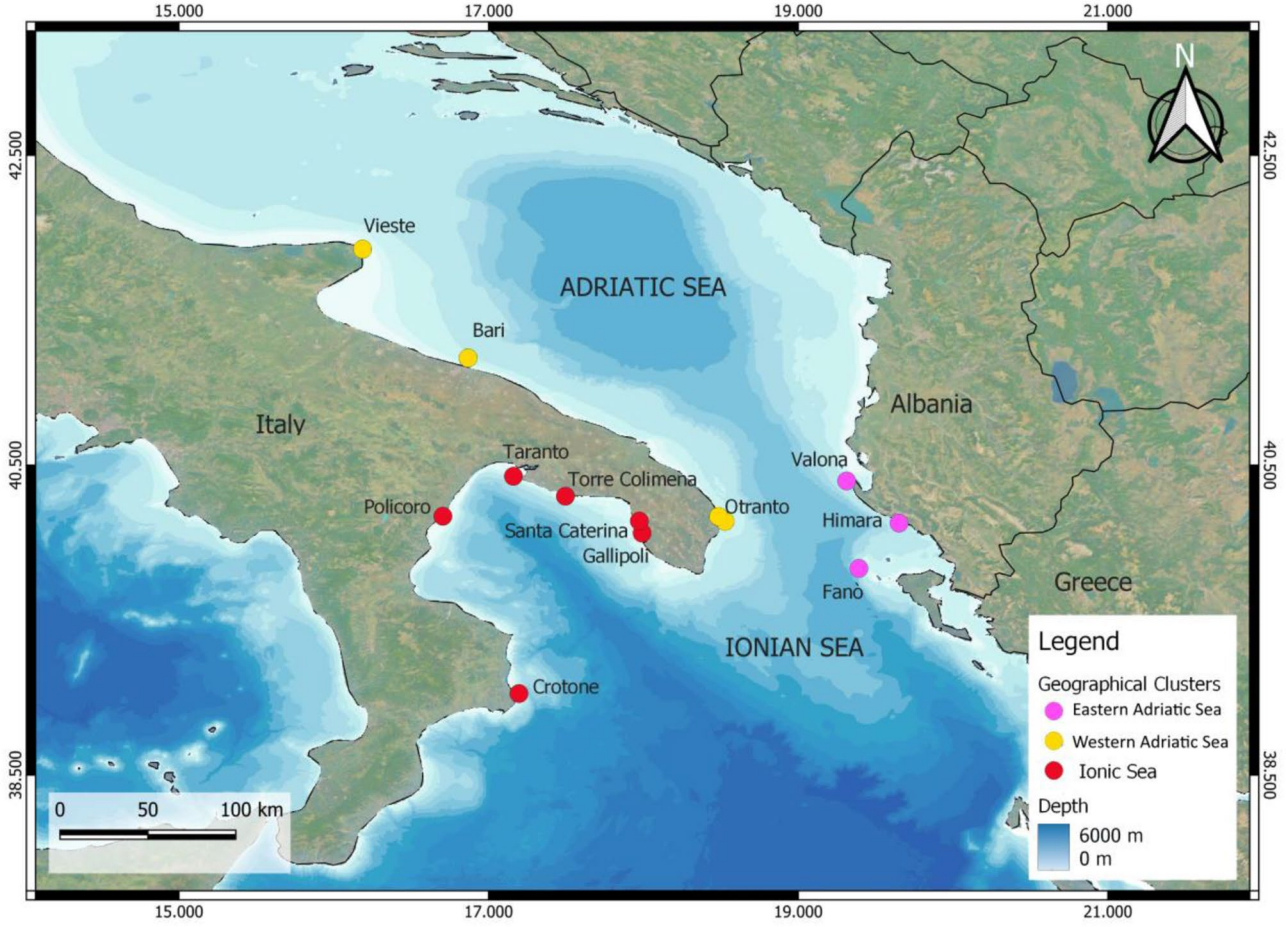
Study area and sampling spots (dots) of the Mediterranean monk seal eDNA monitoring in Autumn 2022, divided by geographical clusters. Pink: Eastern Adriatic Sea, yellow: Western Adriatic Sea, red: Ionian Sea. Made with qGIS 3.22.8

The study area includes the Southern Adriatic Sea and the Northern Ionian Sea, located in the central Mediterranean Sea, including the Gulf of Taranto (Italy) and the Otranto Channel (Italy, Greece, Albania) (Fig. 1). This region was ideal for the study as it is data-deficient, with reported sightings and prior studies already detecting eDNA [13]. Local volunteers were recruited during the summer of 2022 using the GFM social network. Nineteen people, of whom fourteen were Italian and five Albanian, joined the project. As shown in Fig. 1, thirteen sampling spots were selected based on volunteer availability and suitable seal reproductive habitats, such as rocky coastlines with marine caves [5].

Some volunteers collaborated as a team at the same location, either assisting each other during the procedures or dividing tasks to complete the sampling process more efficiently. All volunteers were regular sea users for either business or leisure reasons (e.g., working for NGOs, protected areas, or diving centers, or engaging in recreational activities such as kayaking or sailing) which allowed them to regularly collect samples near the coast using either kayaks, stand-up paddles, sailing, or motor boats during their sports or work activities. Volunteers may have had no prior education or experience in research activities (74%), or they may have worked with conservation organizations, universities or in protected areas (26%).

As recommended by Tweddle et al. (2012 [33]), volunteers received in-person training, including information on seal conservation, presentation of eDNA, its use for species monitoring, and how to collect and filter eDNA samples. We also distributed one sampling kit for each sampling spot during the training, with written instructions. Two video tutorials on the sampling and filtering processes outlined in the training session were also available during the entire study period, should volunteers need additional guidance.

### Volunteers in the field

Volunteers were involved in eDNA collection and filtration from September 15th to December 15th, 2022. They were instructed to collect samples on five specific dates, approximately once every three weeks. These dates were the same for all sampling spots, with a few days of flexibility allowed to accommodate bad sea conditions or personal constraints.

The water collection procedure adhered to the established protocol from previous research such [12, 13, 34], consisting of the extraction of 12 L of surface water using bilge pumps to push water into disposable Bag-inBoxes containers. During the water collection, volunteers recorded data such as date and hour, GPS coordinates, and any issues encountered, such as collecting a lower volume of water due to practical constraints. To minimize the risk of contamination between consecutive samples, volunteers sterilized with a 10% bleach solution the reusable bilge pump after each sampling and, once on the sampling spot, they rinsed it with seawater several times before each new sampling.

**Fig. 2:**
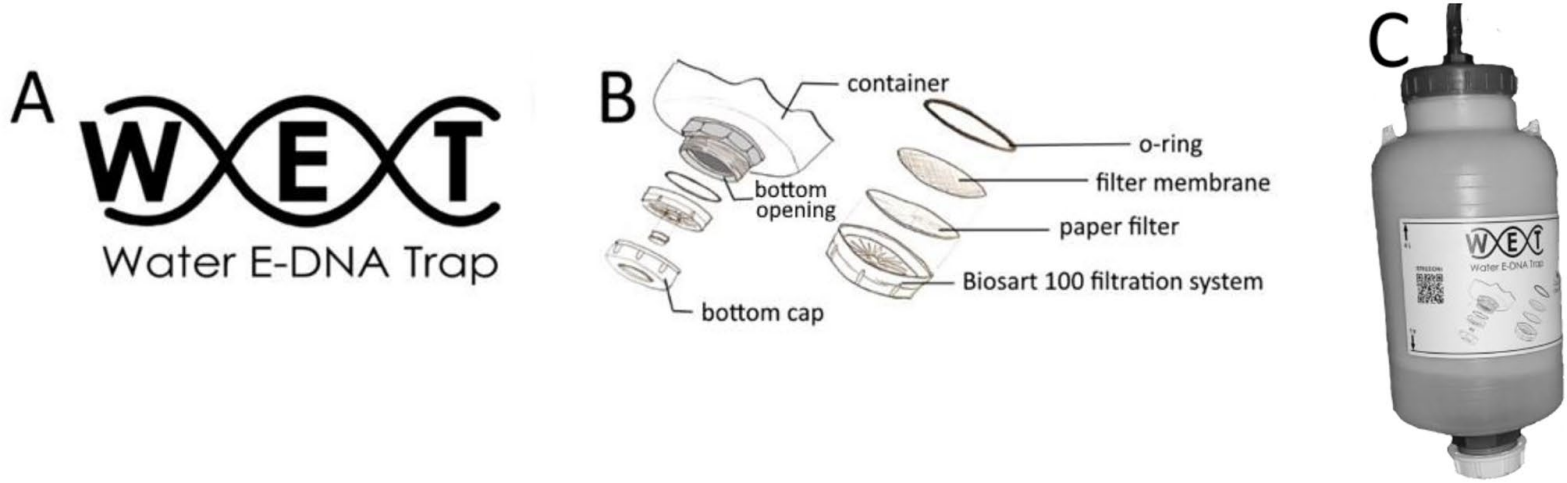
The WET system: Logo (**A**), detail of filtration system (**B**), overview (**C**)

The water collected into the Bag-in-Boxes was then filtered following the same protocol, thus filtering each sample in three 0.45 μm pore cellulose nitrate filter membranes (one membrane every four litres). However, the WET system was used instead of the previously used vacuum pump system (Fig. 2A). The WET can be used in the field without electricity, using a manual pump instead of an electric pump. It consists of a 4-litre filtering container with an opening adapted to accommodate a filter support (modified from a BioSart 100 filtration system, Sartorius) (Fig. 2B) and an opening on the opposite side that connects to a pump (Fig. 2C). The main difference with the traditional system is that water is forced to pass by the filter membrane by increased positive pressure in the container instead of the negative pressure created by the vacuum pump. Filtration time varied based on the applied pressure and water composition, ranging from 30 min to several hours for each sample. During the filtering, volunteers took note of the date of filtration, the time needed to complete the filtration for each filter, and any observations of problems encountered. The reusable equipment, such as the WET container, was sterilized using a 10% bleach solution after each sample was filtered.

Always following the protocol, once filtering was over, filters were wrapped in aluminum paper and plastic bags to avoid contamination, labelled, and stored in a freezer to avoid the development of mould and bacteria until transported to the lab for analysis. The filters were then transported in ice by volunteers or by priority mail and reached the MarHE Center Lab of the University of Milano-Bicocca in January 2023, where the lab analysis (DNA extraction and real-time PCR) was conducted following the standard procedure.

### Monk seal eDNA lab analysis

Extractions were performed, one for each filter, including equipment blanks, using the DNeasy^®^ PowerSoil^®^ Kit (Qiagen) as per the manufacturer’s protocol. PCR working solutions consisted of 25 µl of H2O and 25 µl of DNA. To prevent cross-contamination, the workspace and equipment were cleaned with bleach before each extraction.

Quantitative real-time PCRs (qPCRs) were performed using the MarVer2 locus, identified as the most effective for seals due to its high detectability, species specificity, short sequence length (71 bp), and 100.7% qPCR amplification efficiency [12]. Each replicate included 2 µl of eDNA extract, 0.1 µl of [10 µM] MarVer2 primer solution, 5 µl of Bio-Rad SsoFast EvaGreen Supermix with Low ROX, and 2.8 µl of Milli-Q water. The qPCRs were run using a Step One Plus (BIO-RAD) system with cycling conditions: 95 °C for 10 min (initial denaturation), 40 cycles of 95 °C for 15 s (denaturation), and 56 °C for 1 min (annealing and extension). Each eDNA extract was run in triplicate, with positive (216 ng/µl seal tissue DNA) and negative (no-template) controls included.

eDNA detection outcomes from the qPCR were divided into negative and positive signals. Negative signals (NS) indicated no amplification, while positive signals included both seal DNA detectable but not quantifiable (DBNQ) and positive quantifiable detection (PQD). DBNQ corresponded to a copy number between the limit of detection (LOD, Ct = 40) and the limit of quantification (LOQ, Ct = 35.8), while PQD indicated a copy number above the LOQ. Positive detection was defined as at least one positive replicate per sample [35]. To confirm species-specific amplification, melting temperature (Tm) analysis of the MarVer2 locus (71.5 ± 0.3 °C) was also applied.

### Assessing accuracy and reliability of CS data

To assess the accuracy and reliability of using CS for eDNA monitoring, we first tested the CS-friendly WET system’s validity compared to a traditional filtration system. An in-lab experiment was conducted to evaluate the WET performance in eDNA detection compared to the traditional vacuum pump filtration system, using 6 L of filtered marine water spiked with the seal’s eDNA. The spike was done by soaking 1.5 g of seal tissue in 50 ml of marine filtered water inside a Falcon tube for three hours. Subsequently, the spiked 50 ml water was poured into a 12-litre Bag-in-box filled up with marine filtered water. Then, 1.5 L of spiked water were filtered with the two systems using both 22 μm and 45 μm filter membranes, for a total of four filtrations, two for each system. Finally, PCRs were run with ten replicates for each 22 μm filter and three replicates for each 45 μm filter, using a Step One Plus (BIO-RAD) qPCR with the following temperature cycling conditions: 95 °C for 10 min (initial denaturation), 95 °C for 15s (40 denaturation cycles), 56 °C for 1 min (annealing and extension). Each of the eDNA extracted by the filter membranes was run in together with negative (no-template) controls. The difference between the two systems was assessed by comparing the percentage of positive samples found in each system. We also quantified the WET malfunctioning probability by calculating percentages of technical problems reported by volunteers during filtration. Technical problems were divided into: loss of water, filter breakage, WET cap breakage, and manual pump breakage. We also assessed the risk of contamination in the WET system performing a negative control, as suggested by Rees et al. [11]. We asked a volunteer to filter tap water (negative control) on a WET previously used to filter a sample suspected to be positive due to a seal sighting that occurred near the sampling location in the same period (and eventually resulted positive). A negative result from such a test would indicate that the sterilization process of the WET system prevents the risk of contamination.

Furthermore, we calculated the percentage of faults made by volunteers during sample collection and filtration. For both, we first compared the effective number of samples or filtrations to the expected number. Filtration faults were further categorized into three types: filters not folded appropriately, samples with more water filtered for a single filter, and samples with incomplete filtration. We also collected reported reasons for faults.

Finally, the detection of any positive eDNA samples was used to evaluate the reliability of both the WET system and the volunteers’ sample processing.

### Questionnaire and score design

A non-identifiable questionnaire was prepared and distributed to every volunteer to assess the social impact of CS coupled with eDNA in seal conservation. The questionnaire aimed at detecting any significant difference before and after being involved in the monitoring campaign of Care4Seals in individual learning outcomes. As some participants did not speak English, the questionnaire was also translated into Italian. Informed consent to participate was given by all participants.

The questionnaire was composed of three main sections exploring pro-monk seal conservation learning outcomes: Perceived Knowledge (10 items), Affective Attitudes (12 items), and Conservation Behaviours (8 items). A demographic section was not included in the questionnaire as it would have made individuals identifiable due to the small population size of the elicited survey. As the questionnaire was only disseminated after the project participation, it followed a retrospective post-then-pre design, which is a popular method to assess self-reported change in knowledge, attitudes, and behaviours in which the before-and-after information is collected only at the end of a participatory program [36]. The retrospective questionnaire offers advantages over the traditional pre-then-post design, such as reducing response-shift bias [37, 38]. However, as a self-report, it may introduce biases like social desirability and subjective accuracy [36]. To mitigate these, we used an anonymous online survey [39–41], provided clear instructions in the Participant Information Sheet (PIS) to encourage honesty [42], and included closed-ended, objective questions with exhaustive, mutually exclusive answers [39].

The first section of the questionnaire (Perceived Knowledge) aimed at detecting any gain in knowledge about monk seal conservation. We designed this section with a ten-item scale on seal knowledge adapted from [43]. As in [44], items included different thematic areas: seal biology (4 items), seal conservation (3 items), and eDNA monitoring (3 items) (Table 1). For each item, volunteers were asked to value their knowledge before and after being involved in the CS project on a four-point, Likert-type scale (0-no knowledge, 1-low knowledge, 2-moderate knowledge, 3-high knowledge), similar to other retrospective questionnaires [38, 45]. The individual score corresponded to the sum of the answers’ points [42], here ranging from 0 to 30 for the entire seftion (0 to 3 for each item, Table 1).

**Table 1:** Items of the perceived knowledge section from the questionnaire for citizen scientists of the project Care4Seals in Autumn 2022 in the Adriatic-Ionian region, divided into subsections by thematic areas and with possible score values

Item	Topic	Subsection	Score
1	Mediterranean monk seal distribution	Seal biology	0–3
2	Mediterranean monk seal sightings in your area	Seal biology	0–3
3	Mediterranean monk seal habitat	Seal biology	0–3
4	Mediterranean monk seal reproductive period	Seal biology	0–3
5	Guidelines to correctly behave when encountering a monk seal	Seal conservation	0–3
6	Human-related risk of extinction of the Mediterranean monk seal populations	Seal conservation	0–3
7	Mediterranean monk seal conservation projects	Seal conservation	0–3
8	Environmental DNA presence, origin, and dispersion in marine water	eDNA monitoring	0–3
9	Scientific monitoring of species based on environmental DNA	eDNA monitoring	0–3
10	Procedures to collect and filter environmental DNA samples	eDNA monitoring	0–3

The second section aimed at detecting any change in affective attitudes - i.e. the feelings, beliefs, and values held about a scientific object [46], here seal conservation. Affective attitudes were measured by a twelve-item attitude scale including caring value (4 items), interest feeling (4 items), and self-efficacy belief (4 items) (Table 2). The twelve items were statements about beliefs or affective reactions [47] towards the monk seal conservation and were built taking inspiration from other CS studies on attitudes towards bat conservation [48] and bee conservation [24]. Following [47]’s guidelines on assessing attitudes, respondents indicated the extent of their agreement on statements before and after being involved in the CS project on a five-point Likert scale (1-strongly disagree, 2disagree, 3-no opinion or uncertain, 4-agree, 5-strongly agree), and the individual score was defined as the mean across all items after reverse-scoring negative items (Table 2).

**Table 2:** Items of the Affective Attitude section from the questionnaire for citizen scientists of the project Care4Seals in Autumn 2022 in the Adriatic-Ionian region, divided in subsections by type of attitude and with score values. Items that are reversescored are marked with (RS)

Item	Statement	Subsection	Score
11	I care about the monk seals	Caring value	1–5
12	It is important to protect the monk seals	Caring value	1–5
13	Habitats of monk seals should be preserved	Caring value	1–5
14	The importance given to the monk seal conservation is exaggerated (RS)	Caring value	1–5
15	I am interested in the monk seal conservation	Interest feeling	1–5
16	I am indifferent to the poaching of monk seals (RS)	Interest feeling	1–5
17	I want to play an active role in monk seal conservation	Interest feeling	1–5
18	I want to involve other people in monk seal conservation	Interest feeling	1–5
19	My role in the monk seal conservation is negligible (RS)	Self-efficacy belief	1–5
20	I am proud of my role in monk seal conservation	Self-efficacy belief	1–5
21	I often think about how my actions affect the monk seal conservation	Self-efficacy belief	1–5
22	I make the difference for the monk seal conservation	Self-efficacy belief	1–5

The third and last section of the questionnaire aimed at detecting any change in conservation behaviours, i.e. those that aim at minimizing the impact on nature or actively support biodiversity conservation [49]. As for perceived knowledge and affective attitudes, behaviours were self-reported in this study. Self-reporting is the most widely used method to assess conservation behaviours, and there is a significant correlation between self-reported and observed behaviours [50]. Eight conservation behaviours constituting an eight-item scale have been selected by taking inspiration mostly from [44] and adapted for the monk seal conservation (Table 3). Five of them were behaviours affecting the local community, while the others were individual behaviours. Measurement was made using a five-point frequency Likert-type scale for each behaviour (1-hardly ever, 2-occasionally, 3-sometimes, 4frequently, 5-almost always), and the individual score was calculated by the mean across all items, similarly to [44].

**Table 3:** Items of the conservation behaviours section from the questionnaire for citizen scientists of the project Care4Seals in Autumn 2022 in the Adriatic-Ionian region, with indication of the subsection (individual or with direct influence on the local community) and score values

Item	Behaviour	Subsection	Score
23	I dedicate time to the conservation of the monk seal	Individual	1–5
24	I donate or spend money on the conservation of the monk seal	Individual	1–5
25	I speak about monk seals with my family and friends	Community	1–5
26	I speak about monk seals with colleagues or clients	Community	1–5
27	I spread awareness about monk seal conservation to my local community	Community	1–5
28	I speak about the importance of monk seal protection with people in my community	Community	1–5
29	I read about monk seal conservation	Individual	1–5
30	I vote for candidates that support environmental protection and human-wildlife coexistence	Community	1–5

### Questionnaire quality control

The quality of the questionnaire was assured by a three-step validating process which included literature review, expert feedback, and stakeholder feedback, following [51]’s framework for evaluating CS projects. The literature review is detailed above for each questionnaire’s section. The expert feedback was made by sharing drafts with the research team and additional experts in natural science questionnaires [23]. The stakeholder feedback consisted of a field test from at least 8 individuals similar to the study population [23], which in this case, were people who had a secondary role in the activities such as assistance in sampling and filtering or transport of samples. Moreover, consistently with other studies [24, 43] we evaluated the internal reliability and coherence of the scales by calculating the Cronbach’s Alpha index, which is a measure of the internal consistency of a scale [52].

### Questionnaire statistical analysis

Before analysis, we merged data from English and Italian versions of the questionnaire on a unique database on IBM^®^ SPSS^®^ Statistics 27.0.1, transforming answers from the Likert scale into numbers corresponding to the single-item score. All the statistical analysis and graphs were carried out using SPSS. For each section and subsection, we calculated individual scores for “before” and “after” participating in Care4Seals. We then used descriptive statistics on individual scores to describe the level of perceived knowledge, affective attitudes and conservation behaviours of volunteers before and after being involved. In the Perceived Knowledge section, we considered a score below 15/30 (< 50%) as insufficient knowledge, between 15/30 and 21/30 ( > = 50% and < 70%) as sufficient knowledge, and above 21/30 ( > = 70%) as good knowledge. In the Affective Attitudes and Conservation Behaviours sections, we considered a score of < 2.5/5 as a negative attitude/infrequent behaviour, >=2.5/5 and < = 3.5/5 as a neutral attitude/average behaviour and > 3.5/5 as a positive attitude/frequent behaviour. Then, we calculated the mean among all scores for all sections and subsections both before and after to test if they were significantly different. As [53] found the t-test more robust than the Wilcoxon signed-rank test for Likerttype data and small samples, even when non-normal distributed, we used t-tests to compare scores before and after each section and subsection, regardless of normality. Finally, we calculated the percentage of increase (Perceived Knowledge) and improvement (Affective Attitudes and Conservation Behaviours) between before and after in each section and subsection.

## Results

### Comparison between traditional and WET filtration systems

Table 4 summarises the results of the comparative test on eDNA yield obtained using the WET filtration system and the traditional vacuum pump filtration system. No difference was found between the two systems in detection probability, as the detection ratio (number of positive replicates over total replicates) was 1 (100% of positive replicates) in both systems and with both 0.22 μm and 0.45 μm filter membranes.

**Table 4:** qPCR results from the experiment comparing eDNA detection using a traditional vacuum pump filtration system (VP) and the newly-developed WET system (WET). Ct: cycle threshold; TM: melting temperature

System	Filter porosity (µm)	Mean Ct (SD)	Mean TM °C (SD)	% of positive replicates
WET	0.22	21.65 (0.16)	71.78 (0.07)	100% (10 out of 10)
VP	0.22	20.43 (0.59)	71.89 (0.10)	100% (10 out of 10)
WET	0.45	21.54 (0.10)	71.84 (0.09)	100% (3 out of 3)
VP	0.45	20.49 (0.16)	71.83 (0.09)	100% (3 out of 3)

The newly developed CS-friendly WET filtration system’s technical problems are reported and quantified in Table 5. Loss of water was recorded in 5.8% of filtrations, the filter broke in 1.2% of filtrations, the bottom cap of the WET in 1.8% of filtrations, and 5.0% of manual pumps broke. The control samples (tap water) used to assess the contamination risk associated with the use of the reusable WET system all tested negative.

**Table 5:** Malfunctioning encountered with the WET filtration system during the eDNA monitoring of Mediterranean monk seal in Autumn 2022 in the Adriatic-Ionian region

Type of malfunctioning	Number of failures over total number	Percentage of malfunctioning	Reason(s) of malfunctioning
Loss of water	10/171	5.8%	Gasket defects, incorrect position of the bottom cup
Filter breakage	2/171	1.2%	Excessive pressure
WET cap breakage	3/171	1.8%	Excessive pressure
Manual pump breakage	1/19	5.0%	Poor quality pumps, excessive usage

### Reliability of sample processing by citizen scientists

Due to personal constraints of volunteers, not all sampling spots were covered on each scheduled date, resulting in a total of 60 samples collected over the study period, instead of the expected 65 from the 13 sampling locations (92%). Additionally, due to volunteer faults or malfunctioning of the system described above, not all samples were filtered in triplicate. As a result, 168 filtrations were performed instead of the expected 180 (93%) - plus three filtrations from the negative control, resulting in a total of 171 filtrations. Moreover, volunteers committed faults during the filtration: 12.2% of the filters were not folded appropriately, 6.7% of samples were mistakenly filtered with 12–8 L per filter (instead of 4), and volunteers did not complete the filtration in 5.0% of the samples (Table 6).

**Table 6:** Quantification of faults made by volunteers during the filtration of eDNA samples for Mediterranean monk seal monitoring in Autumn 2022 in the Adriatic-Ionian region

Type of fault	Number of faults over total number of filters (F) or samples (S)	Percentage of faults	Reason(s)
Filters not folded appropriately	22/171 F	12.9%	Volunteer’s forgetfulness or misunderstanding
Samples with more water filtered for a single filter	4/60 S	6.7%	Volunteer’s forgetfulness or misunderstanding
Samples with incomplete filtration	3/60 S	5.0%	Personal time constraints or loss of filters

Finally, of the 60 samples analyzed for the seal’s eDNA, 27 (45.0%) tested positive – of which 25 (92.6%) with at least one PQD replicate – indicating detectable levels of the target species.

### Social impact

**Fig. 3:**
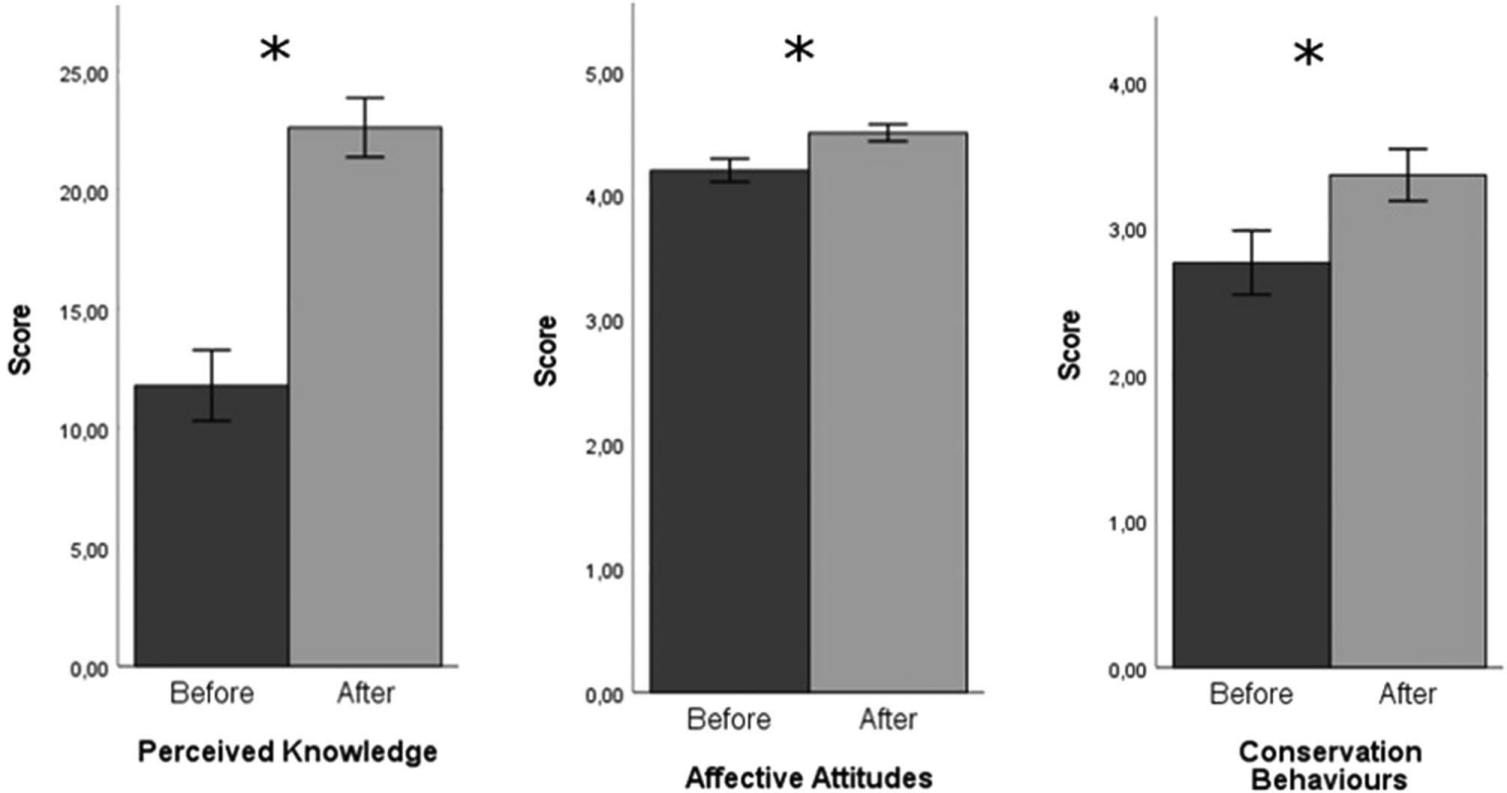
Scores of the three sections of the retrospective post-then-pre questionnaire given to volunteers before (dark grey) and after (light grey) taking part in Care4Seals eDNA monitoring for the conservation of Mediterranean monk seal in the Adriatic-Ionian region. Asterisks indicate a significant difference (t-test). Error bars represent +/-1 Standard Error with a 95% Cl. Made with IBM^®^ SPSS^®^ Statistics 27.0.1

All nineteen volunteers involved in Care4Seals completed the retrospective post-then-pre questionnaire (100% response rate). Concerning the internal scale reliability, the Cronbach Alpha resulted in 0.929 for the Perceived Knowledge, 0.788 for the Affective Attitudes, and 0.945 for Conservation Behaviours. All three sections of the questionnaire presented a significant difference before and after being involved in Care4Seals (Fig. 3). Indeed, results from the t-test with a 95% confidence interval show that the mean score was significantly different before and after in the Perceived Knowledge section (*p* < 0.001, + 92%), Affective Attitudes section (*p* < 0.001, + 7%), and Conservation Behaviours section (*p* < 0.001, + 22%) (Table 7). Significant differences were also found in each subsection of the questionnaire: +57% in the perceived knowledge of seal biology (*p* < 0.001), + 71% in the perceived knowledge of seal conservation (*p* < 0.001), + 225% in the perceived knowledge of eDNA monitoring (*p* < 0.001), + 5% in the caring value (*p* < 0.001), + 7% in the interest feeling (*p* < 0.001), + 9% in the self-efficacy belief (*p* = 0.002), + 26% in individual conservation behaviours (*p* = 0.001), and + 20% in community conservation behaviours (*p* = 0.001) (Table 7).

**Table 7:** Score means before and after and results of the t-test in the sections (bold) and subsections (italics) of the questionnaire given to volunteers involved in Care4Seals eDNA monitoring for the conservation of Mediterranean monk seal in the Adriatic-Ionian region. Data from the statistical analysis made with IBM^®^ SPSS^®^ Statistics 27.0.1

Section/subsection	Mean (SD) before	Mean (SD) after	df	t	*p*	Improvement
**Perceived knowledge**	**11.80 (6.50)**	**22.63 (5.42)**	**18**	**-8.577**	**< 0.001**	**+ 92%**
*Seal biology*	*5.53 (2.65)*	*8.68 (2.73)*	*18*	*-6.352*	*< 0.001*	*+ 57%*
*Seal conservation*	*4.16 (2.27)*	*7.11 (1.52)*	*18*	*-6.564*	*< 0.001*	*+ 71%*
*eDNA monitoring*	*2.11 (2.71)*	*6.84 (1.77)*	*18*	*-7.877*	*< 0.001*	*+ 225%*
**Affective attitudes**	**4.20 (0.41)**	**4.50 (0.30)**	**18**	**-4.630**	**< 0.001**	**+ 7%**
*Caring value*	*4.38 (0.47)*	*4.62 (0.45)*	*18*	*-4.256*	*< 0.001*	*+ 5%*
*Interest feeling*	*4.28 (0.48)*	*4.59 (0.41)*	*18*	*-4.652*	*< 0.001*	*+ 7%*
*Self-efficacy belief*	*3.93 (0.65)*	*4.30 (0.51)*	*18*	*-3.684*	*0.002*	*+ 9%*
**Conservation behaviours**	**2.76 (0.95)**	**3.36 (0.77)**	**18**	**-3.972**	**< 0.001**	**+ 22%**
*Individual*	*2.30 (0.99)*	*2.89 (0.76)*	*18*	*-3.869*	*0.001*	*+ 26%*
*Community*	*3.04 (1.01)*	*3.64 (0.86)*	*18*	*-3.828*	*0.001*	*+ 20%*

**Fig. 4:**
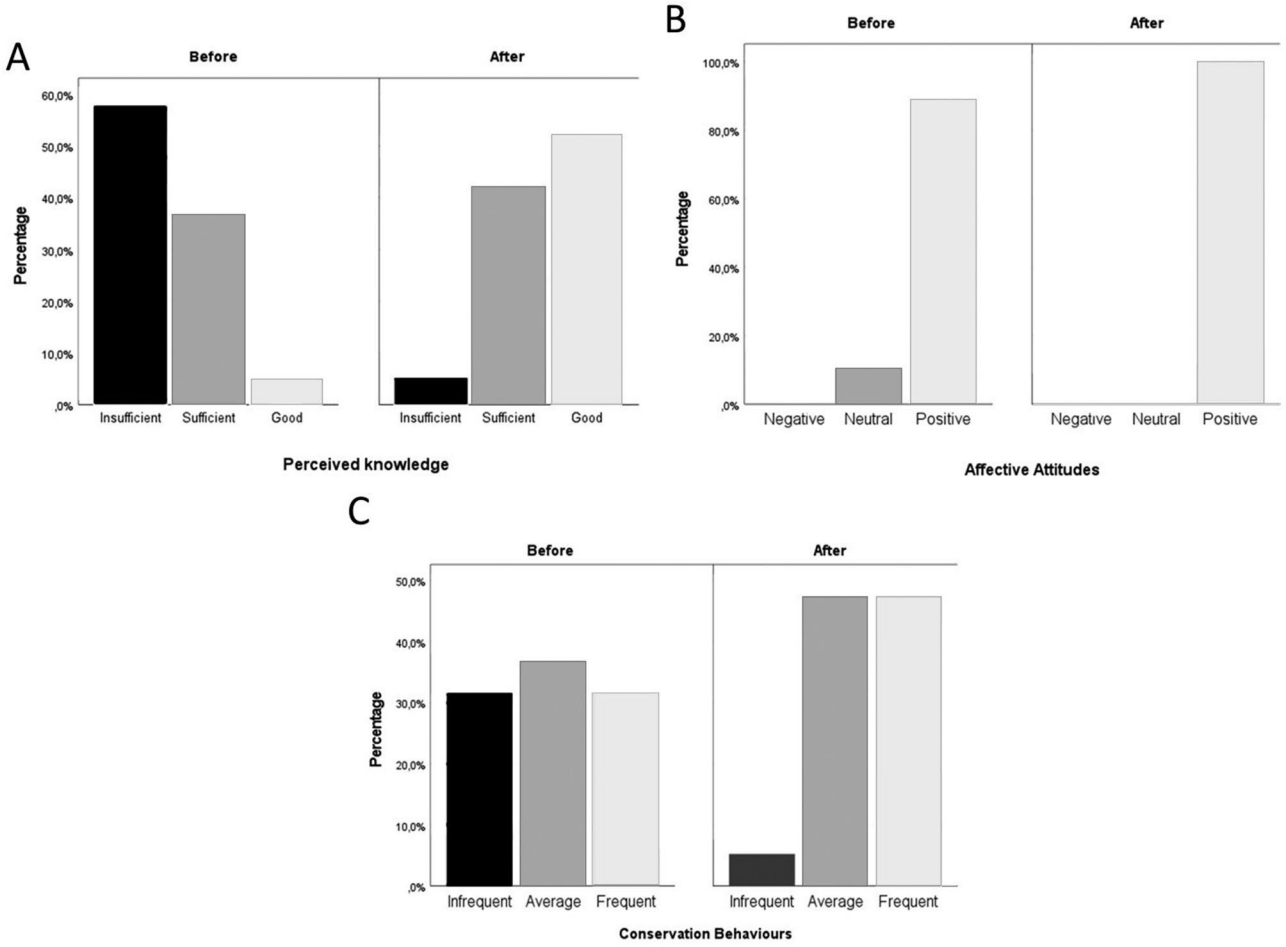
Percentage of volunteers’ perceived knowledge (**A**), affective attitudes (**B**) and conservation behaviours (**C**) levels before and after being involved in Care4Seals eDNA monitoring for the conservation of Mediterranean monk seal in the Adriatic-Ionian region. Insufficient knowledge: <50%; sufficient knowledge: >=50% and < 70%; good knowledge: >=70%. Negative attitude: <2.5; neutral attitude: >=2.5 and < = 3.5; positive attitude: >3.5. Infrequent behaviour: <2.5; average behaviour: >=2.5 and < = 3.5; frequent behaviour: >3.5.Made with IBM^®^ SPSS^®^ Statistics 27.0.1

Results show that 57.9% of the volunteers (11/19) perceived to have insufficient knowledge, 36.8% (7/19) sufficient knowledge, and 5.3% (1/19) good knowledge on seals before being involved in Care4Seals. After being involved, 52.6% (10/19) perceived to have good knowledge, 42.1% (8/19) sufficient knowledge and 5.3% (1/19) insufficient knowledge (Fig. 4). Before being involved in CS, almost all volunteers (89.5% corresponding to 17/19) already had positive affective attitudes, only 10.5% (2/19) had neutral attitudes, and none had negative attitudes towards seals. After taking part in Care4Seals, all of them (100%) had positive affective attitudes towards seals (Fig. 4). In regards to the individual scores before being involved in Care4Seals, 31.6% of volunteers (6/19) presented infrequent conservation behaviours, 36.8% (7/19) average conservation behaviours, and 31.6% (6/19) frequent conservation behaviours. Instead, after being involved, 47.4% (9/19) presented frequent conservation behaviours, 47.4% (9/19) average conservation behaviours, and only 5.3% (1/19) infrequent conservation behaviours (Fig. 4).

## Discussion

### Robustness of eDNA data obtained through citizen science

The first objective of this study was to assess the scientific validity of using CS for eDNA monitoring, in particular filtration of water samples as previous volunteers involved in Spot the Monk eDNA collection never processed the seawater samples. In this study, we tested on a large scale the prototype of a low-cost device designed for simple and autonomous eDNA filtration (without electricity), called WET.

The WET performance in eDNA detection was tested in the lab in comparison with the vacuum pump system, and both had a detection ratio of 1, which confirms other studies [30, 54] suggesting that there is little or no difference between different filtration systems and methods. Regarding the WET malfunctioning, 5.8% of the filtrations in this study presented a loss of water from the bottom cup, which can potentially lead to loss of eDNA. Moreover, excessive pressure broke the filter in 1.2% of filtrations causing loss of one filter per sample, the bottom cap of the WET in 1.8% of filtrations, and one manual pump broke by usage. As it was the first time the WET was used on a large scale, malfunctioning should have been expected. Learning from this study experience, improvements to the WET system should be made to overcome defects, such as adding an extra valve to prevent excessive pressure that can break the filter or the cap and using more resistant manual pumps. Overall, percentages of malfunctioning were relatively low, and thus not of relevance for the eDNA results.

With regard to the risk of contamination, cross-contamination between different locations was avoided as each location was equipped with a different WET system. Contamination could still be possible between consecutive samples if the equipment is not well sterilized. Strict contamination controls are essential for eDNA to be reliably taken for species distribution monitoring [55], avoiding the possibility of false positives. For this reason, we included in our study an equipment negative control. It could be argued that the WET system is more difficult to sterilize as it has a large water container with folds and furrows, but the contamination test was reassuring: negative control samples loaded in the same WET where positive samples were collected, were all tested negative for monk seal eDNA. This suggests that if thorough equipment cleaning is carried out between each sampling, then cross-sample contamination can be avoided. Thus, preventing contamination relies on volunteers’ attention in cleaning the equipment. It would be advisable to add a negative control before each filtration. Nevertheless, this approach would require more time and budget as each sample would consist of four filters instead of three.

Due to volunteers’ constraints, only 60 samples were collected instead of the expected 65 (92%). While this reduced the spatial coverage, the study would not have been possible without the volunteers, and the fact that some spots were not sampled on every date is still preferable to not sampling at all. Additionally, 168 filtrations were completed instead of the planned 180 (93%) due to volunteer faults and WET malfunctions. While these issues may have affected the consistency of eDNA results and reduced the likelihood of detecting more positives, the overall impact on the study’s conclusions is likely minimal, given the small deviation from the planned protocol.

Despite having been trained to filter eDNA samples and having free access to video-tutorials and written instructions with pictures in each kit, some volunteers committed the following faults during the filtration process:

1. Not folding the filter (12% of filters): this can bring the loss of eDNA and thus increases the chances of false negatives.
2. Forgetting to change the filter (6.7% of samples): filtering more litres per filter makes them not quantitively comparable with other samples – but they still can be used to detect eDNA.
3. Not completing the filtration for personal time constraints (5.0% of the samples): they obtained 2 filters (8 L) instead of 3 (12 L) per sample, which limits eDNA yield and, thus, detectability [56], besides adding inhomogeneity in the sample set.

The low percentage of faults made by volunteers—similarly to system malfunctions—suggests a negligible impact on eDNA detection. Notably, although most of these faults and malfunctions likely reduced the chances of detecting eDNA, the results showed that 45% of the samples tested positive for the target species in a low-density area, with a high proportion of quantifiable detections (92.6%). Such a detection rate is robust under these conditions and aligns with previous studies, such as [13], which reported 42.2% positive detection in similar conditions using only researcher-handled samples filtered through traditional methods.

Overall, these findings highlight that the combination of the WET system and volunteer-supported sample collection and filtration is a reliable approach for eDNA-based monitoring, though there is room for improvement. The WET used in this study was a prototype, and its limitations could only be identified through a large-scale trial. With these results, we now have sufficient data to address its structural weaknesses, which can be improved in subsequent versions. Future studies using citizen science for eDNA sampling should build on these lessons, incorporating enhanced training protocols where volunteers not only observe procedures but also practice them under researcher supervision. Regular reminders during the study period could further minimize procedural faults and improve overall reliability.

Finally, it is important to note that false negatives are likely to occur for various reasons, including the presence of the target species without its eDNA being successfully detected or its rapid degradation due to environmental factors [11]. For instances, marine currents play a fundamental role in the dispersion of biological material, thus affecting eDNA detection. It is difficult to quantify to what extent, though, since their effects vary from case to case and depends on meteorological/oceanographic conditions. It must be kept in mind, however, that, if on the one hand the signal can be diluted and dispersed more or less quickly, new molecular traces are continuously released into the water column, replacing the old or too diluted signals that have traveled too far from the point where they were released. These challenges are inherent to eDNA studies, regardless of whether they are citizen science-driven or researcher-led. While a positive eDNA detection (in the absence of contamination) confirms the species’ presence, a negative result does not necessarily indicate its absence. This limitation highlights the importance of collecting samples from the same area across different time periods to enhance detection reliability and account for natural variability in eDNA availability—a goal that can be more feasibly achieved through citizen science initiatives.

### Social aspect of citizen science for eDNA monitoring

Monk seal conservation efforts mainly focus on ecological aspects, although social viewpoints are much more relevant as threats to the species are linked to human interactions [7]. This study also aimed to assess the effectiveness of a retrospective questionnaire in evaluating the social impact of conservation science (CS) and explore whether combining CS with eDNA techniques could enhance public knowledge, attitudes, and behaviors toward seals. The questionnaire used in this study had a limited sample size (19 participants) compared to similar studies ranging from 60 [44] to more than 950 participants [57]. Small sample sizes reduce statistical power, increasing the likelihood of failing to detect significant differences or overestimating observed effects [58]. Therefore, while differences were identified, they should be interpreted with caution, even with a 100% response rate from a representative sample of volunteers.

Findings for the Perceived Knowledge section suggested that people involved in the project significantly increased their knowledge of the study object, supporting other studies finding an increase in both perceived [24, 44] and actual knowledge [42, 44, 48] of volunteers involved in CS or ecotourism initiatives. The overall knowledge improvement was of 92%. Interestingly, all three subsections increased conspicuously, but the most impressive increase was in the eDNA subsection (+ 225%), confirming the role of CS in spreading scientific literacy [59] and, consequently, critical thinking [60]. Critical thinking empowers citizens to engage in decision-making on sciencebased social issues [61], such as conservation. The increased knowledge in the seal conservation subsection (+ 71%) proves that CS can be used to spread awareness on conservation issues, especially considering that knowledge may influence attitude change and conservation behaviours [62].

The Affective Attitudes section was the one recording the smallest change (+ 7%). That is not surprising as people taking part in CS initiatives may already have positive environmental attitudes [42], leading to the ceiling effect, which is the difficulty in measuring changes in a response scale when participants are initially at an extreme of the scale [24]. It could be suggested that this was the case in this study, as, before participating in the project, 89.5% of participants had positive attitudes towards seals. Similarly, statistically significant but relatively low attitude change was found in other studies [24, 44, 48, 63], while others did not even find significant differences [42]. However, participation in CS initiatives can also reinforce existing pro-conservation attitudes [64]. All three subsections of Affective Attitudes significantly improved, supporting previous studies on the role of CS in enhancing caring value [65], interest and self-efficacy belief [66, 67]. The Self-efficacy Belief subsection had the highest improvement (+ 9%), which is extremely important for seal conservation considering the self-efficacy principle of conservation psychology, according to which people are more likely to actively participate in conservation when they feel confident to make a positive impact [68]. Self-efficacy belief is indeed a core component of motivation [67] and is positively correlated with pro-conservation behaviours [69]. The result, therefore, suggests that taking part in CS initiatives, such as eDNA monitoring, makes people feel empowered and thus more prone to conservation actions.

The last section of the questionnaire registered a significant overall improvement on Conservation Behaviours. Even if previous studies on the learning outcomes of CS or ecotourism found low [25, 70] or no [71] improvement in conservation behaviours, the significant increase in conservation behaviours of volunteers involved in the field, here marine activities, supports [49], claiming that nature activities contribute largely to engagement in conservation behaviours. Eventually, conservation behaviours are of extreme importance as nature conservation can only be achieved with human behavioural change [72]. Moreover, the Community Behaviours, including behaviours influencing the entire local community, such as discussing seal conservation with family, friends, or clients, or voting for political candidates with pro-conservation programs, showed a 20% improvement. This finding suggests the potential for CS projects to influence not only participants but also people around them, promoting both individual and community engagement, as widely recognized [73, 74]. However, it is important to note that these conclusions are based on a limited sample size with an indirect measurement, which warrants cautious interpretation. Despite this limitation, the results are valuable as they contribute to addressing a gap in the assessment of community behaviours or collective actions, which have rarely been studied in previous CS projects [73], and have predominantly relied on researcher reflection [74].

Overall, while the findings from the questionnaire suggest a potential change in perceived knowledge, attitudes, and behaviours towards monk seal conservation from citizen science participants, they should be seen as indicative rather than conclusive due to the small sample size. Importantly, this study validates the retrospective questionnaire as a tool for assessing social impact, which, with the involvement of more volunteers in the future, could be used to reliably measure these changes on a broader scale.

### Future perspectives

The participants in this study already had predominantly positive attitudes toward seals. Future studies could replicate this approach by involving individuals with neutral-to-negative attitudes toward seals, such as fishermen and other stakeholders who directly or indirectly impact seal conservation (e.g., tourists and tourism operators). This would allow for a comparison of questionnaire results across different categories of stakeholders and an exploration of whether their background influences their attitudes and behaviours. For instance, specific conservation behaviours, such as direct interactions with seals, could be included to better assess changes in behaviours relevant to fishermen and other groups. Such studies would provide valuable insights into how citizen science initiatives could engage diverse audiences and foster conservation efforts among groups with varying baseline attitudes. We strive to underline the importance of involving the new generations in this type of program, considering that the beneficial effect of greater involvement in environmental and conservation issues has an exponentially greater and longer-term effect if the message is imparted upstream. It is with this idea that GFM promotes many educational activities aimed at the younger generations.

## Conclusion

The results of this study showed that CS is a scientifically valuable tool for the eDNA monitoring of monk seals. Although it may need refinements and improvements and despite its limitations, such as faults made by volunteers, CS allowed the collection of valuable data on a large spatiotemporal scale.

This study also validated the use of a retrospective questionnaire as an effective tool for assessing the social impact of CS initiatives for eDNA monitoring of the seal. While the small sample size necessitates cautious interpretation of the significant differences observed, the results suggest that directly engaging local communities not only enhances ecological monitoring but also addresses key social dimensions of monk seal conservation. Participants reported improvements in perceived knowledge, attitudes, and conservation behaviors related to seals, including actions influencing their local communities such as voting for political candidates with pro-conservation programs. Importantly, since participants were regular visitors to the sea for leisure or work, their increased awareness could be particularly valuable for conservation efforts, though further research with a larger sample is needed to confirm these findings.

## Electronic Supplementary Material

Below is the link to the electronic supplementary material.

Supplementary Material 1

Supplementary Material 2

## Data Availability

Data is provided within the manuscript or supplementary information files.

## Abbreviations

CS: Citizen Science
EDGE: Evolutionary Distinct and Globally Endangered
eDNA: Environmental DeoxyriboNucleic Acid
GFM: Gruppo Foca Monaca APS
PCR: Polymerase Chain Reaction
WET: Water eDNA Trap
