# Correction: Citizen science in eDNA monitoring for Mediterranean monk seal conservation

**DOI:** 10.1186/s12862-025-02348-0

**Published:** 2025-01-07

**Authors:** Sofia Bonicalza, Elena Valsecchi, Emanuele Coppola, Valeria Catapano, Harriet Thatcher

**Affiliations:** 1 Department of Biomedical Sciences, University of Edinburgh, Edinburgh, UK; 2 Gruppo Foca Monaca APS, Via Carlo Emery 47, 00188 Rome, Italy; 3 Department of Environmental and Earth Sciences, University of Milano-Bicocca, Piazza della Scienza 1, 20126 Milan, Italy

**Correction:** ***BMC Ecol Evo*** **24, 148 (2024)**

10.1186/s12862-024-02338-8

Following publication of the original article [1], the authors reported an error in one of the author’s name, where Valeria Capatano should be corrected to Valeria Catapano.

The original article [1] has been updated.
